# Notice of the Meeting of the State Medical Society

**Published:** 1854-05

**Authors:** 


NOTICE.

The next annual meeting of the "Iowa State Medical and Chirurgical Society," will be held at Muscatine, Wednesday, June 14th, 1854.

JOHN H. RAUCH,
Recording Secretary.

By a reference to the proceedings of the A. M. A., found in the preceding pages, it will be seen that by a resolution unanimously adopted, the profession in those States in which no State organization exists, is urged to a speedy formation of societies. Not only State Societies but also county organizations, and we take this occasion to urge upon our brethren, the propriety of organizing thoroughly. Let us not lag in the rear of other States, but as we are in the line of rapid progress in all other departments, we should not allow this important element of true greatness to languish for want of interest.

We hope to meet, and to greet too, many of our warm and generous medical friends at the above meeting at Muscatine, not only those already members, but also those who have not yet become such.—We have received the proceedings of a great many of the other States around us, which from their nature and character, plainly show a lively interest in their organizations. Pennsylvania boasts of a county or district society which not only publishes its proceedings, but an excellent Journal also. So also in Virginia there is a Society which publishes a very spirited and useful periodical.

